# Efficiency of Original versus Generic Intravenous Iron Formulations in Patients on Haemodialysis

**DOI:** 10.1371/journal.pone.0135967

**Published:** 2015-08-31

**Authors:** Maria Luisa Agüera, Alejandro Martin-Malo, Maria Antonia Alvarez-Lara, Victoria Eugenia Garcia-Montemayor, Petra Canton, Sagrario Soriano, Pedro Aljama

**Affiliations:** 1 Servicio de Nefrología. Hospital Universitario Reina Sofía, Córdoba, Spain; Instituto Maimónides de investigación biomédica de Córdoba (IMIBIC), Universidad de Córdoba, Córdoba, Spain; RedInRen, Instituto de salud Carlos III, Spain; 2 Dialysis Unit, SOCODI SL, Córdoba, Spain; The Pennsylvania State University Hershey Medical Center, UNITED STATES

## Abstract

**Aims:**

The appropriate use of intravenous (IV) iron is essential to minimise the requirements for erythropoiesis-stimulating agents (ESAs). The clinical efficacy of generic IV iron compared to the original formulation is controversial. We evaluated the changes that were induced after switching from a generic IV iron to an original formulation in a stable, prevalent haemodialysis (HD) population.

**Methods:**

A total of 342 patients were included, and the follow-up period was 56 weeks for each formulation. Anaemia parameters and doses of ESA and IV iron were prospectively recorded before and after the switch from generic to original IV iron.

**Results:**

To maintain the same haemoglobin (Hb) levels after switching from the generic to the original formulation, the requirements for IV iron doses were reduced by 34.3% (from 52.8±33.9 to 34.7±31.8mg/week, p<0.001), and the ESA doses were also decreased by 12.5% (from 30.6±23.6 to 27±21μg/week, p<0.001). The erythropoietin resistance index declined from 8.4±7.7 to 7.4±6.7 IU/kg/week/g/dl after the switch from the generic to the original drug (p = 0.001). After the switch, the transferrin saturation ratio (TSAT) and serum ferritin levels rose by 6.8%(p<0.001) and 12.4%(p = 0.001), respectively. The mortality rate was similar for both periods.

**Conclusions:**

The iron and ESA requirements are lower with the original IV iron compared to the generic drug. In addition, the uses of the original formulation results in higher ferritin and TSAT levels despite the lower dose of IV iron. Further studies are necessary to analyse the adverse effects of higher IV iron dosages.

## Introduction

Patients with chronic kidney disease (CKD) stage 5D on haemodialysis (HD) have increased mortality rates. This mortality risk is partly related to the severity of the patients’ anaemia, which has been associated with adverse clinical outcomes. Low haemoglobin (Hb) levels have been associated with higher mortality[1], increased cardiovascular events and reduced health-related quality of life in HD patients. However, randomised controlled trials have shown that normal Hb values are not associated with better survival [2, 3]. Therefore, the adequate management of anaemia is considered to be one of the most important factors in treating HD patients.

Anaemia, a common complication of CKD [4], has a complex pathogenesis and mainly arises due to inadequate erythropoietin production, insufficient iron storage, inflammation, vitamin B12 and folic acid depletion, severe secondary hyperparathyroidism and blood loss during the HD procedure. In CKD patients undergoing HD, anaemia was treated with blood transfusions prior to the introduction of recombinant erythropoiesis-stimulating agents (ESAs). However, recent clinical trials have reported controversial outcomes with regards to higher haematocrit levels [1–3]as well as high doses of ESAs[5, 6]. The Hb target level has consequently been lowered, decreasing the need for high doses of ESAs.

The use of intravenous (IV) iron to treat renal anaemia is currently increasing [7]. Under conditions of renal anaemia, the administration of iron is essential because absolute and functional iron deficiencies are common [8]. Because iron deficiencies must be corrected in order to increase Hb levels, moderate doses of ESAs should be administered to achieve the desired Hb levels [9–10]. However, the excessive and indiscriminate use of IV iron preparations can have insidious and serious adverse consequences, including iron overload [11], cardiovascular disease, immune deficiency and a potential increase in the risk of microbial infections[12].

The mainside effects of IV iron are ascribed to the abrupt rise in serum levels of non-transferrin-bound iron (NTBI) [13]. NTBI causes the formation of reactive oxygen species (ROS), which damage the endothelium, promote endothelial dysfunction and cause inflammation [14]. Moreover, IV iron can lead to neutrophil [15] and monocyte dysfunction[16, 17]. The formulation of the administered iron is important for these effects [18]; in a previous study [17], we showed that a generic version of iron elicits a greater increase in ROS, intercellular adhesion molecule (ICAM)-1 and apoptosis than the original formulation.

Cost minimisation relies on the principle of substituting the original drugs with therapeutically equivalent alternatives and expecting equal outcomes at a reduced cost[19]. Generic drugs are usually less expensive than the original compound but must demonstrate bioequivalence[20]in order to be approved by regulatory authorities [21]. However, not all groups have shown that these products are clinically interchangeable with the original formulations[22].

Few studies have analysed the clinical outcomes after switching from the original formulation of IV iron to a generic drug. However, Rottembourg et al.[23] observed a reduction in Hb levels in patients who had been switched from the original to the generic IV iron formulation.

In April 2011, our HD centres switched the original formulation of IV iron-sucrose to a generic drug for economic reasons, and patients were followed to monitor for adverse events. Unexpectedly, in June 2012 (13 months later), a change in the price of the original formulation prompted hospital administrators to revert back to the original IV iron formulation. Thus, we had the opportunity to prospectively follow patients who used the original IV iron formulation for an additional 13 months.

The principal aim of our study was to examine our prospectively collected data to analyse the effects of switching from generic IV iron-sucrose to the original formulation in a stable, prevalent population undergoing HD.

## Materials and Methods

### Study design

This observational and longitudinal repeated-measures study compared the same measurements that were collected prospectively before and after a switch from a generic IV iron to an original IV iron in a cohort of stable HD patients.

**Fig 1:**
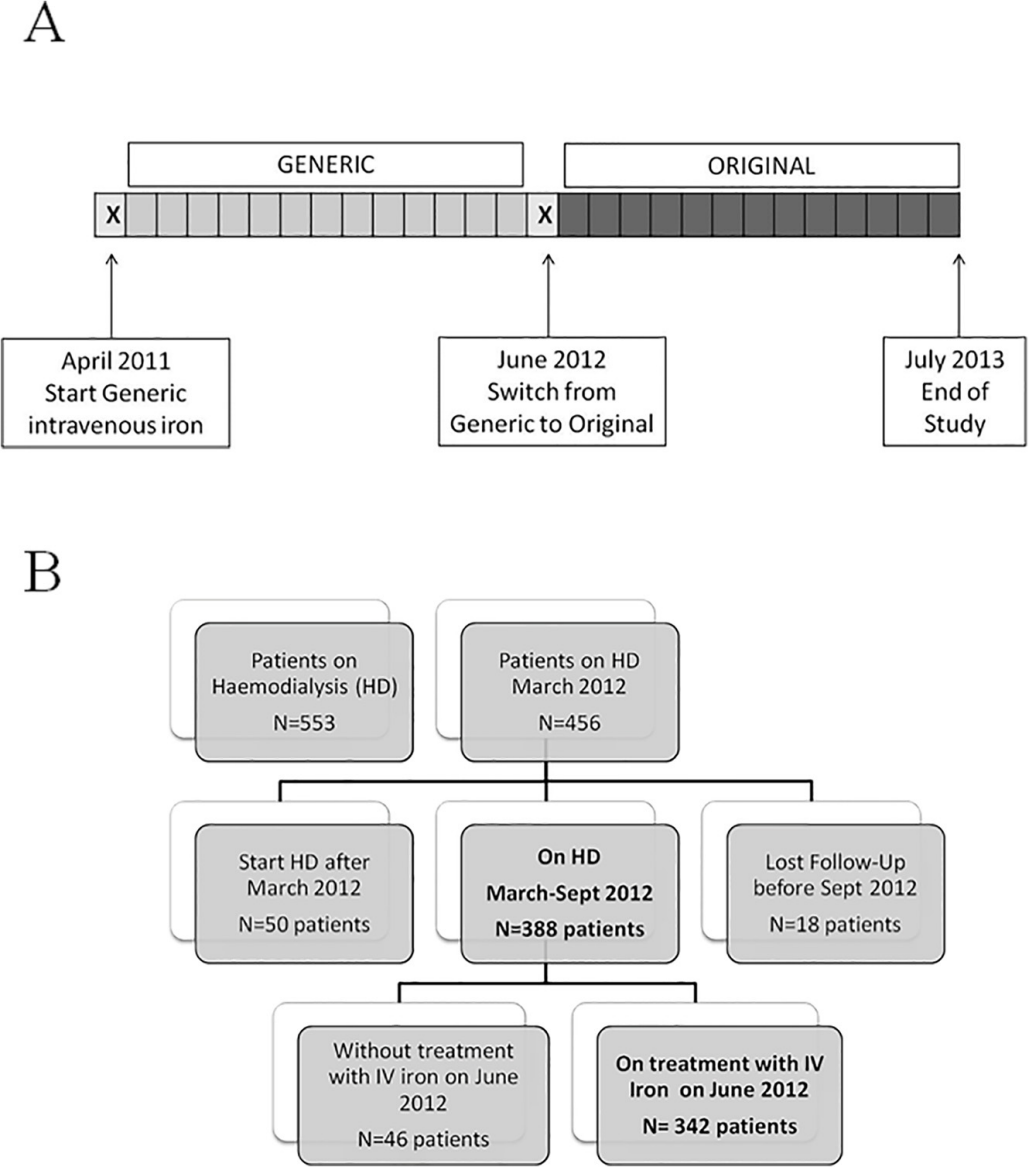
Study design (1A) and algorithm of study population selection (1B).

The study protocol is shown in Fig 1A. Beginning in April 2011, all of the patients were treated with the same generic formulation of IV iron along the study. In June 2012, the IV iron was changed to the original formulation and patients were followed for an additional 13 months. The data obtained from April 2011 to June 2012 were excluded because both medications may have been simultaneously available at the centre. The study was approved by the local ethics committee (Comité de la ética de la Investigación de Córdoba, Consejería de Salud; ref. 2645), and signed informed consent was obtained from patients before their inclusion in the study.

### Study population

All of the prevalent, stable patients from our 7 HD centres from April 2011 to July 2013 were selected. All patients were dialysed using ultrapure dialysate fluid (<1 bacterial colony-forming unit/ml and <0.03 endotoxin units/ml). Residual renal function declined throughout the study, from 8.8% in the generic period to 5.6% in the original period (p = 0.031). However, weekly dialysis doses in terms of hours/week were similar during the 2 treatment periods (12.2 ± 0.15 in generic period vs. 12.5 ± 0.19 hours/week). Most patients were dialyzed 3 times per week, and the percentages of patients were similar between periods (92.1% of the population in generic period vs. 91.3% in original period). Eligible patients (Fig 1B) were those who treated with IV iron for at least 3 months before the formulation was switched (June 2012). The patients were censored if they received a kidney graft or died during the follow-up.

### Treatment of anaemia: IV iron/erythropoiesis-stimulating agents

IV iron (generic or original) was administered during the HD session at a frequency ranging from once per month to up to twice per week. The ESA used was darbepoetinalfa, which was given intravenously during the mid-week dialysis session. The doses of IV iron and darbepoetinalfa were recorded at the beginning of each month throughout the 26 months of the study and were expressed as a weekly dose (mg per week for IV iron and μg per week for darbepoetinalfa). The doses of IV iron and ESA were adjusted for nightly to maintain the target Hb levels.

### Study variables

The serum Hb, packed cell volume (PCV), ferritin levels, transferrin saturation ratio (TSAT), C-reactive protein (CRP) levels, adequacy of dialysis (eKt/V) and dry body weight were assessed monthly. Blood samples were collected before the dialysis session. If the participant had repeated measurements of any of these variables within a single month, the first measurement was used in the analysis. Theerythropoiesis resistance index (ERI) was calculated as the ratio between the ESA dose, expressed in international units (translation rate 1:200) per kg body weight per week (IU/kg/week), and the Hb level (g/dl).

### Statistical methods

The continuous data were expressed as the means ± standard deviation (SD) and median and interquartile range (IQR) for normal and skewed distributions, respectively. The categorical data were expressed as percentages. The data were logarithmically transformed when appropriate. Between-period comparisons (generic versus original period) were performed using the paired Student’s *t*-test and repeated-measures analysis of variance (ANOVA) for continuous data and either the McNemar test (χ^2^) or Cochran’s Q test for categorical data when appropriate. Statistical analyses were performed with Statistical Package for Social Sciences (SPSS) 12.0. Two-sided P values of less than 0.05 were considered statistically significant.

## Results

### Study population

Of the 553 prevalent adult patients on HD in Cordoba from April 2011 to July 2013, 342 were eligible for inclusion (Fig 1B). The study population was 59% male, with a mean age of 70±16 years. The main causes of end-stage renal diseases were diabetes (68 patients, 20% of the study population), hypertension/vascular disease (37 patients, 11%) and glomerulonephritis (34 patients, 10%). The average dialysis duration was 89±71 months (Table 1).

**Table 1:** Baseline demographic and clinical characteristics of the study population (N = 342 patients).

Age, years (mean ± SD)	70 ± 15.9
Male, n (%)	202 (59%)
Primary Renal Disease, n (%)	
-Unknown	120 (35.1%)
-Diabetes	68 (19.9%)
-Hypertension	37 (10.8%)
-Glomerulonephritis	34 (9.9%)
-Tubulointerstitial Nephritis	24 (7%)
-APKD	24 (7%)
-Other Nephropathies	35 (10.2%)
Vintage of HD, months (mean ± SD)	89.5 ± 71.3
BMI, Kg / m2 (mean ± SD)	26.5 ± 6.1
Charlson Index (mean ± SD)	5.9 ± 2.3
-Diabetes Mellitus, n (%)	76 (22.3%)
-Myocardial Infarction, n (%)	37 (11%)
-Heart Failure, n (%)	44 (13%)
-Liver Disease, n (%)	10 (3.2%)

Of the 342 study subjects, 271 remained on HD by the end of the study (79%), 55 patients died (16%) and 16 patients received a kidney transplant (5%) during the 26 months of follow-up. There was no significant difference in the mortality rate between periods (p = 0.183), and no adverse events were associated with switching from the generic to the original IV iron formulation.

### Treatment of anaemia: IV iron/ESA dose

**Fig 2:**
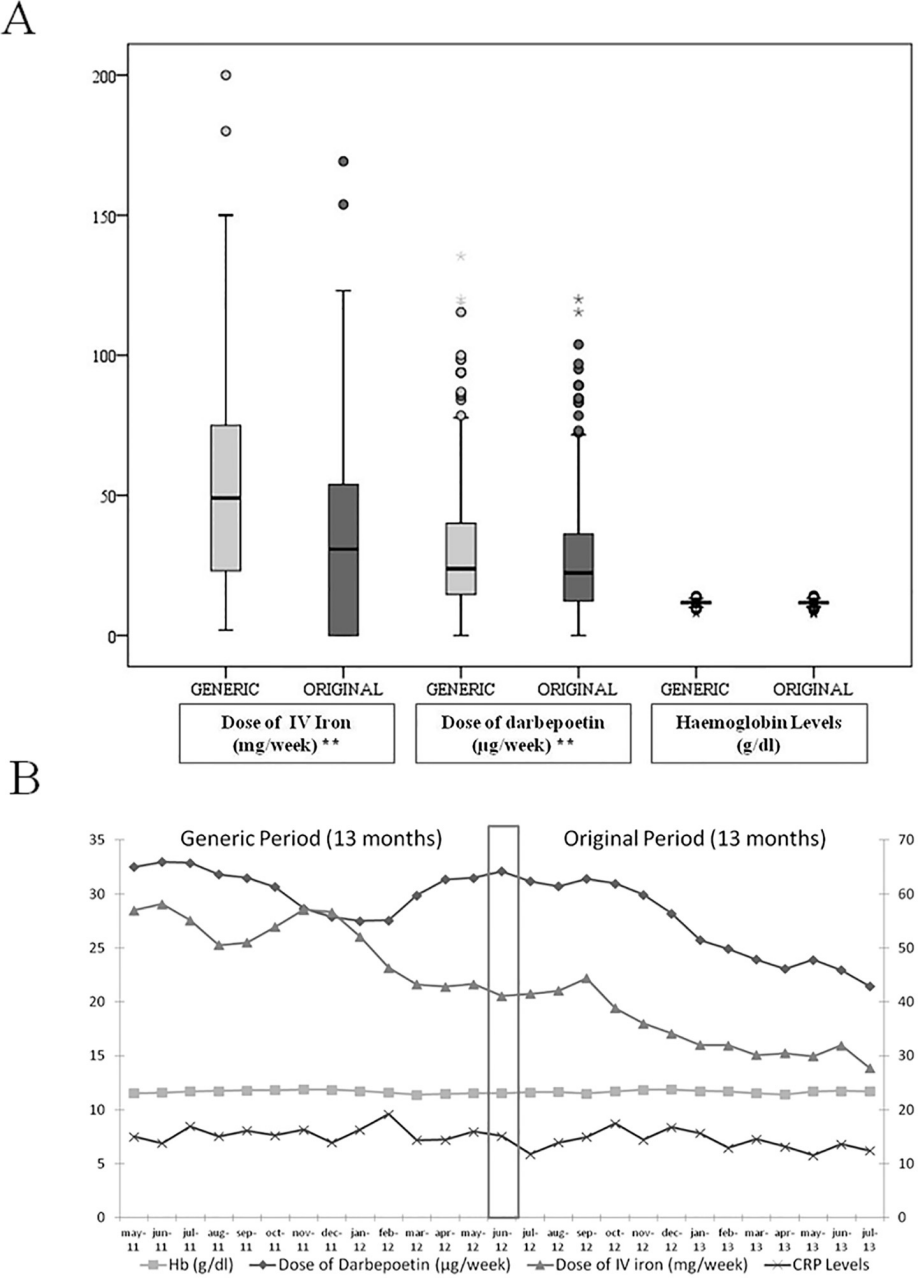
Haemoglobin, intravenous iron and darbepoetin doses in each period (2A) and monthly (2B).

The mean dose of IV iron per patient was 52.8±33.9 mg/week during the Generic period and 34.7±31.8 mg/week during the Original period (p<0.001, Fig 2A), representing a 34.3% decrease. The mean dose of ESA was 30.6±23.6 μg/week during the Generic period and 27±21 μg/week during the Original period (p<0.001, Fig 2A), representing a 12.5% reduction.

### Parameters related to anaemia: Hb, ERI, TSAT and ferritin levels

The Hb levels were stable throughout the study (26 months). The mean Hb level was 11.6±0.8 g/dl during the Generic period and 11.6±0.9 g/dl during the Original period (p>0.5, Fig 2A). The monthly data for IV iron dose, ESA dose and Hb levels throughout the study are shown in Fig 2B.

**Fig 3:**
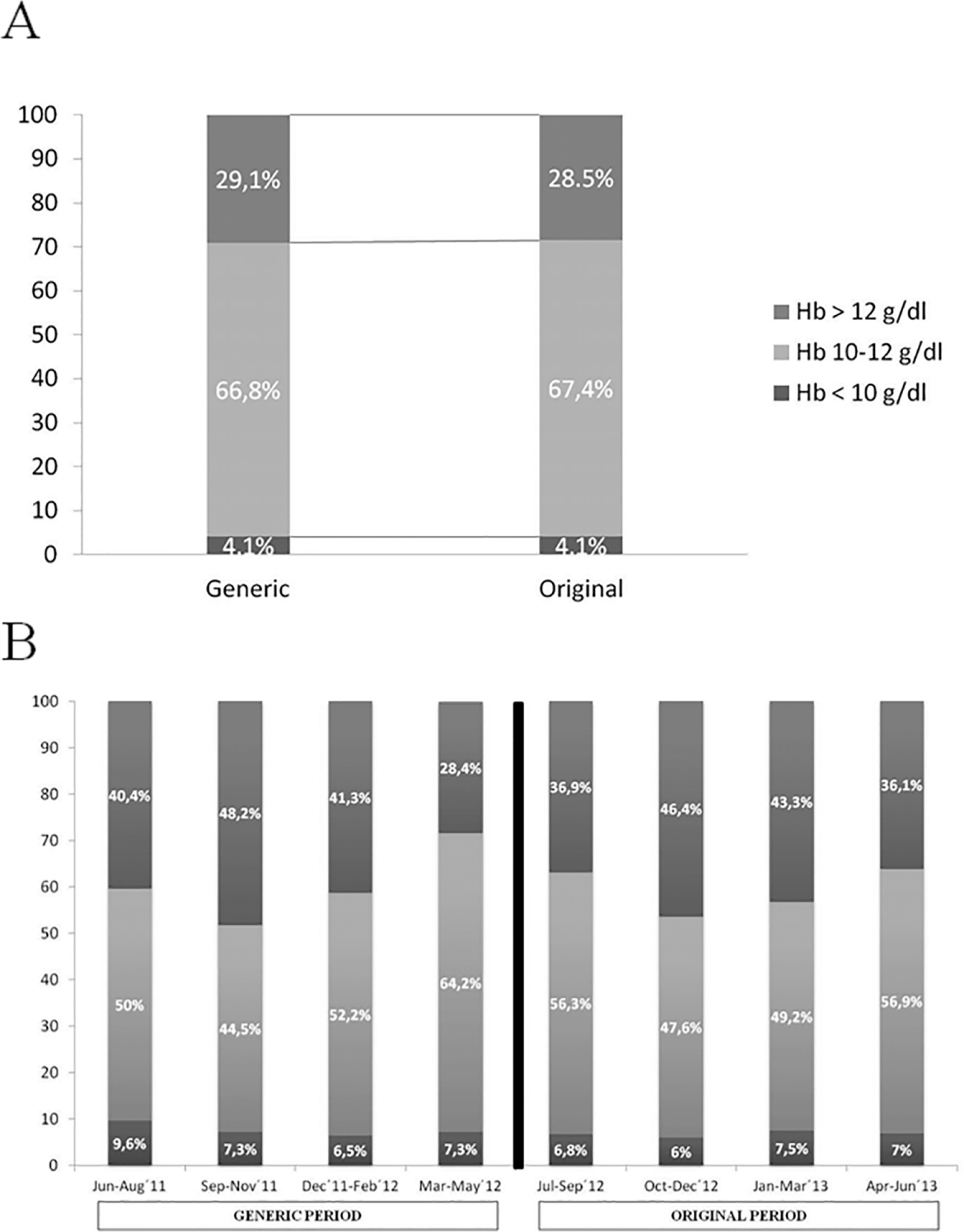
Variability in haemoglobin in each period (3A) and in quarterly (3B).

The variability in Hb, which was based on the distribution of the population within the 3 groups (below the target range, Hb<10 g/dl; within the target range, Hb = 10–12; or above the target range, Hb>12 g/dl)[24], remained stable throughout the study. In both periods, 4% of patients had mean Hb levels below 10 g/dl (Fig 3A). In the quarterly study, there were no significant differences between quarters (Fig 3B). The PCV was also stable throughout the study.

**Fig 4:**
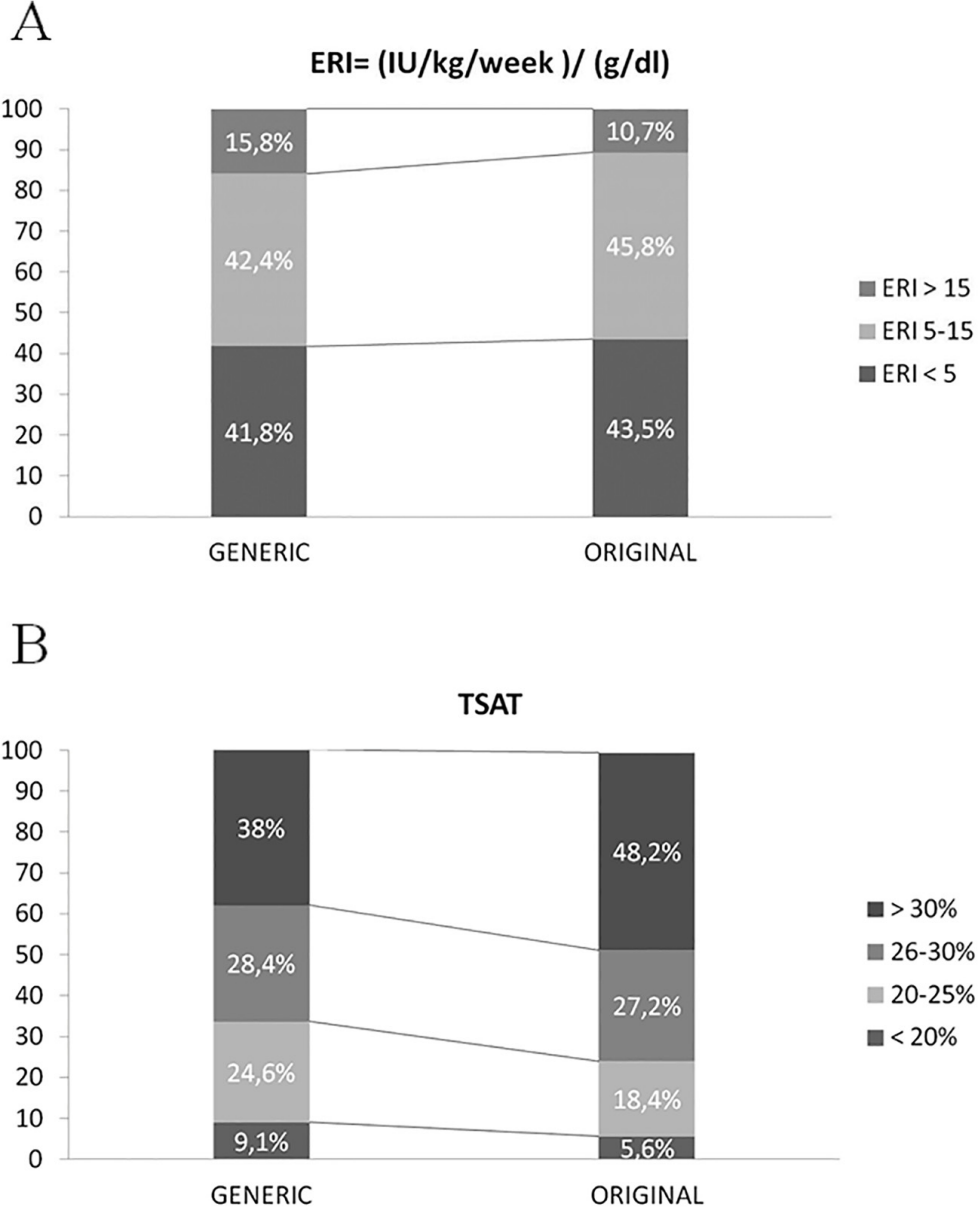
Distribution of the population depending on ERI (4A) or TSAT levels (4B).

The mean ERI level was 8.4±7.7 IU/kg/week/g per 100ml during the Generic period versus 7.4±6.7 IU/kg/week/g per 100ml during the Original period (p = 0.001), which constituted a 12% decrease. Based on the ERI level, the study population was divided into 3 groups: <5; between 5 and 15; and >15 (Fig 4A). 15.8% of patients had an ERI>15 during the Generic period compared with 10.7% during the Original period (p<0.001).

The mean TSAT level increased by 6.8% between the Generic and Original periods (28.6±7.2% and 30.7±7.6%, respectively) (p<0.001). Based on the TSAT levels, the study population was divided into 4 groups: <20%; 20% to 25%; 26% to 30%; and >30% (Fig 4B). More patients were in the TSAT>30% group during the Original period than during the Generic period (48.2% versus 38%, respectively; p<0.001).

The serum ferritin levels also rose between the Generic and Original periods. The median ferritin levels (IQR) were 507 (338) mg/dl during the Generic period and 579 (393) mg/dl during the Original period (12.4% increase) (p = 0.001). These data were confirmed after the logarithmic transformation of the ferritin levels (data not shown).

### Other variables

There were no significant differences in the dialysis doses (expressed as the bicompartimentaleKt/V), frequency and duration of HD sessions, type of dialysis membrane, number of blood transfusions, serum CRP and albumin levels, or any other parameters between the Generic and Original periods (data not shown).

## Discussion

The administration of the original formulation of IV iron to a prevalent HD population achieved the same Hb levels as the administration of the generic drugwhile using 34.3% less IV iron and a 12.5% lower ESA dose. Despite the lower dose of IV iron, the levels of ferritin and TSAT increased after the switch from the generic to the original IV iron formulation. These results suggested that a change from the generic to the original iron formulation can be made safely as long as the doses are adjusted according to the characteristics of the new formulation.

WHO guidelines promote the use of generic drugs as a strategy to mitigate high pharmaceutical prices [19]. Because generic and original drugs must demonstrate bioequivalence, one would expect that switching formulations would not be associated with any significant change in everyday clinical practices[21]. After a retrospective analysis of 2,070 single-dose clinical bioequivalence studies that were submitted to the United States Food and Drug Administration (FDA) from 1996 to 2007, Davit et al. [22] confirmed that the actual bioequivalence from the generic to original product is less than 10%. A meta-analysis by Kesselheim et al. [25]found that brand-name (original) drugs for cardiovascular disease were not superior to generic drugs. However, though no meta-analyses are available for other types of drugs (such as immunosuppressants[26] and antiepileptic medications), most studies have reported poorer results from the use of generic drugs compared to the original formulations.

For IV iron, few studies have previously compared the generic formulations to the original formulations in HD patients. Nonetheless, Rottembourg et al. [23]used partial retrospective data obtained before and after the switch from the original to the generic formulation to compare the clinical and economic results of the two formulations. Our group has previously measured oxidative stress and apoptosis in blood samples from patients who were treated with various iron compounds, including the generic and original iron-sucrose formulations [16]. Other groups have also developed a rat model to compare the effects of these formulations in vitro [27]. These studies suggest that generic compounds are more deleterious than the original versions.

The IV iron is used in patients on HD to treat renal anaemia. The current recommendation for renal anaemia treatment in patients on HD is the use of the lowest doses of IV iron and ESA necessary for achieving the desired target Hb level on an individual basis[9, 10]. In this study, switching from generic to original IV iron lowered the doses of both drugs (IV iron and ESA) without compromising Hb target levels during an ample (13 months) period of observation. The lower requirements for IV iron and ESA using the original IV iron formulation versus the generic formulation suggests that the former has a greater potency, which should be taken into consideration when analysing the outcomes and costs of the different treatments.

Patients on maintenance HD receive numerous therapeutic interventions, although the clinical benefits of these treatments are often uncertain. Since their development, ESAs have generally been used to avoid the need for blood transfusion. However, an association between the use of high doses of ESA and increased mortality has recently been reported[5,6]. At present, the use of IV iron in combination with the lowest possible dose of ESA in controlling renal anaemia is increasing [28], thoughthe association between IV iron and serious adverse events [29] and mortality [30] remains unclear. Szczech et al.[31] suggested that using IV iron to reduce ESA doses could have important cardiovascular benefits, while Zitt et al.[32]found that the use of IV iron was associated with a 22% reduction in mortality. Moreover, Kalantar-Zadeh et al.[33] found that the risk of death was lower for patients on dialysis who received <400 mg of IV iron per month compared to patients receiving no IV iron. However, Feldman et al.[34] did not confirm an association between IV iron and mortality using techniques that account for changes in iron dosing and morbidity over time and Kshirsagar et al [35] did also not find any association of IV iron to increased mortality. More recently, other authors[36,37]have confirmed a similar mortality between patients on HD without iron and patients receiving “low” doses of iron. Both studies suggested an association between “high” doses of iron and mortality, describing high-dose of IV iron as 300 mg/month [36] or 1050 mg/3 months [37]. A randomised, controlled, open-label study designed to assess the effect of high-dose IV iron on mortality in HD patients (Proactive IV iron therapy in haemodialysis patients, PIVOTAL)[38] will be finished in 2017. In the interim, it seems recommendable to try to lower the dose of IV iron as much as possible. In the present study, we demonstrated a 34% reduction in the dose of IV iron after switching from the generic formulation to the original drug, suggesting that this change may decrease the high mortality rates of patients on HD.

Because the prices of the IV iron preparations were not uniform throughout our study, we could not make an adequate analysis of the costs. However, there was a significant decrease in the doses of ESA and IV iron required for maintaining the same Hb levels. Thus, our data suggest a cost-benefit to using the original formulation of IV iron rather than the generic formulation provided that both formulations have the same price.

The Hb variability responses to ESA may also result in adverse outcomes. We noted similar Hb variability levels[24] during the Generic and Original periods, which were confirmed in the quarterly analysis. During each period, only 4% of patients were below the target Hb range. Another study [23] reported more frequent out-of-range Hb levels when using the generic IV iron formulation versus the original.

Because the Hb levels remained stable and the doses of ESA were progressively reduced, the resulting ERI declined during the Original period. There is no consensus on the cut-off value at which ERI levels increase the mortality of HD patients. Some authors[39, 40]have analysed ERI in quartiles, while Nishio et al.[41] analysed the ERI in tertiles, and Lopez-Gomez et al.[42] divided the population into 3 groups (<5; 5–15; and >15 IU/kg/week/g per 100ml). Regardless of the ERI cut-off, the mortality risk was higher in the patients with increased ERIs. In our study, after 56 weeks of follow-up, the mortality rates were similar between periods.

In our study, the ferritin and TSAT levels increased after the switch from the generic to the original IV iron formulation. Thus, the original IV iron is associated with higher levels of ferritin and TSAT despite being administered at lower doses than the generic formulation. This finding might be attributed to the difference in the kinetics of iron dissociation after changes in the stability of the core of the iron-sucrose complex [43]. Slight alterations in the manufacturing process of iron carbohydrates can result in disparities in the structure, molecular weight distribution and stability of the iron-oxyhydroxide core of the iron-sucrose complex [44]. Morphometric analyses in an animal model have revealed significantly less iron in its physiologically stored form, ferritin, after the administration of the generic formulation versus the original [27]. The deposition of iron in parenchymal tissues instead of the reticuloendothelial system might decrease the amount of iron that is available for effective erythropoiesis [45].

Other variables that could have an effect on anaemia control, such as CRP levels and the frequency and duration of dialysis, were similar between periods. Obviously, there was a possible carryover effect the first 3 months after switching from the generic formulation to original IV iron. However, we felt that the analysis of a long follow-up time (52 weeks) and the exclusion of patients who were lost before the third month could partially overcome this effect. In the statistical analysis, we included the entire study population (N = 342 patients), not just those who remained on haemodialysis at the end of the study (N = 271 patients). When the analysis was repeated for only the 271 patients who were alive on haemodialysis during both treatment periods, the results were similar to that of the entire study population.

The results of this study might be unable to be generalized to other original or generic IV iron formulations. Our recommendation is that the selection of a specific generic formulation should not be based solely on financial considerations, assuming comparable efficacy and safety, because original and generic formulations might not be interchangeable. Our particular generic IV iron formulation had a similar safety profile than the original—the mortality rates and potential clinical side effects were comparable—but disparate efficacy, because we had to adjust the dose of anaemia treatment. Based on our data, we strongly recommended that after changing a generic formulation of IVironto original IV iron, we must be aware of any changes in the parameters of anaemia to make appropriate dose adjustments for the treatment.

The main limitations of this study are inherent to its design. It is not a randomized double blind cross-over study. Although our repeated-measures design allowed us to control for many confounding factors, the data obtained from this observational, single-centre study might not be generalized to the rest of the HD population or to other Generic or Original IV iron formulations. Furthermore, the follow-up period was adequate for analysing the effects of the treatment on anaemia parameters, but it was not valid for evaluatingthe long-term outcomes associated with each formulation. High doses of ESAs[5,6] and IV iron [36, 37] and high ERI levels [39–42] have been linked to increased mortality in HD patients. In this study, we observed that the generic IV iron formulation is associated with higher ESA doses, higher IV iron doses and higher ERIs than the original IV iron formulation. Additional randomised controlled studies with longer follow-up periods must be designed to confirm our results.

In conclusion, the treatment of anaemia in HD patients with the original IV iron formulation requires lower doses of IV iron and ESAs than the generic iron formulation used in this study to achieve greater TSAT and serum ferritin levels and similarly stable Hb levels. Switching from the generic to the original IV iron formulation requires an adjustment in the dose of iron to maintain Hb levels within the target range.
